# Heart in Distress: How Different Shock States Converge on Cardiac Dysfunction

**DOI:** 10.3390/biomedicines14102157

**Published:** 2026-09-24

**Authors:** Georgios E. Zakynthinos, Niki Tripyla, Nikolaos K. Kokkinos, Ourania Katsarou, Ioannis Gialamas, Antonios Lysandrou, Savvas Sarantos, Dionysios Tsoukalas, Evangelos Oikonomou, Gerasimos Siasos

**Affiliations:** 1 3rd Department of Cardiology, Thoracic Diseases Hospital of Athens “Sotiria”, National and Kapodistrian University of Athens, 11527 Athens, Greeceant.lysandrou@hotmail.com (A.L.);; 2 3rd Cardiology Department, “Sotiria” General Hospital of Chest Diseases of Athens, Athina Ioanni Martinou Endowed Chair, Medical School, National and Kapodistrian University of Athens, 11527 Athens, Greece

**Keywords:** shock, cardiac dysfunction, echocardiography, hemodynamics, septic shock, right ventricular dysfunction, fluid resuscitation, vasopressors and inotropes, critical care, point-of-care ultrasound

## Abstract

Shock is traditionally classified according to its primary etiology—septic, hypovolemic, obstructive, or cardiogenic—yet these syndromes frequently converge on a common feature: disturbances of cardiac performance, ventricular loading, and tissue perfusion. Despite distinct initiating mechanisms, altered preload and afterload, ventricular interdependence, vascular dysfunction, maladaptive neurohormonal responses, and, in selected states, impaired myocardial contractility may ultimately reduce effective forward flow and tissue perfusion. Echocardiography provides a rapid, noninvasive bedside tool for characterizing these evolving cardiovascular effects. It enables assessment of left and right ventricular function, cardiac filling, preload responsiveness, pulmonary and systemic venous congestion, fluid tolerance, and broader hemodynamic profiles while facilitating recognition of reversible causes such as cardiac tamponade, acute pulmonary embolism, or severe hypovolemia. This allows clinicians to move beyond static etiologic classifications toward a physiology-based assessment of shock. Beyond diagnosis, echocardiography can inform fluid resuscitation and vasoactive or inotropic therapy. Serial assessment also permits evaluation of therapeutic response and evolving hemodynamic phenotypes. This review synthesizes the mechanisms of cardiac involvement across shock syndromes and highlights echocardiography as a unifying tool for their evaluation and management. By integrating pathophysiology, bedside imaging, and hemodynamic treatment, we propose a physiology-based framework that complements traditional shock classification and may improve diagnostic precision and individualized management in critically ill patients.

## 1. Introduction

Circulatory failure represents a life-threatening condition in which effective blood flow and oxygen delivery become insufficient relative to tissue metabolic requirements and requires rapid recognition and targeted management [1,2]. It is classically categorized into four major forms, cardiogenic, distributive, hypovolemic, and obstructive shock, based on the primary mechanism of hemodynamic impairment. Although this framework remains fundamental in clinical practice, patients frequently present with overlapping features, making precise classification challenging [3,4,5,6].

Beyond primary cardiac causes, alterations in cardiac performance are increasingly recognized as important components of non-cardiac shock states [7]. In cardiogenic shock, primary myocardial or mechanical cardiac dysfunction directly limits cardiac output [8]. In hypovolemic shock, myocardial contractility may initially remain preserved, while reduced venous return and ventricular underfilling critically limit stroke volume. In obstructive shock, cardiac output falls because ventricular filling or ejection is mechanically constrained, as occurs in pulmonary embolism, cardiac tamponade, or tension pneumothorax [7]. In distributive states, profound vasodilation, altered vascular tone, capillary leak, and abnormal blood-flow distribution may coexist with preserved or increased cardiac output, but secondary myocardial dysfunction may develop and substantially modify the hemodynamic phenotype [7].

In sepsis, a dysregulated host response to infection can directly impair myocardial function, leading to sepsis-induced cardiomyopathy, a typically reversible condition characterized by variable systolic and/or diastolic dysfunction involving one or both ventricles [9,10]. Cardiac involvement is common, affecting up to 40–60% of patients with septic shock in the early phase of illness [10,11], and often reflects the global impact of systemic inflammation on both left and right ventricular function. Importantly, shock is a dynamic process in which different mechanisms may coexist or evolve over time [12].

In this review, we examine the mechanisms underlying cardiac involvement across the major shock states, contrasting primary pump failure in cardiogenic shock with secondary, load-dependent, or mechanically mediated cardiac consequences in septic, hypovolemic, obstructive, and anaphylactic shock. We further discuss contemporary diagnostic and therapeutic approaches, the integration of cardiology within multidisciplinary critical care and emergency settings, and the role of early integrated echocardiography in the assessment and management of circulatory failure.

### Review Methodology and Literature Search

This article was designed as a narrative review. A targeted literature search was performed in PubMed/MEDLINE and Scopus for articles published until June 2026. Search terms included combinations of “shock,” “septic shock,” “hypovolemic shock,” “obstructive shock,” “cardiogenic shock,” “anaphylactic shock,” “cardiac dysfunction,” “myocardial dysfunction,” “echocardiography,” “point-of-care ultrasound,” “hemodynamic monitoring,” “right ventricular dysfunction,” “fluid responsiveness,” “vasopressors,” and “inotropes.” Priority was given to international guidelines and consensus statements, systematic reviews and meta-analyses, randomized or prospective clinical studies, large observational studies, and recent high-quality narrative reviews. Experimental and translational studies were included when required to address specific pathophysiological mechanisms, while older seminal studies were retained when they provided foundational physiological evidence. Articles were selected on the basis of direct relevance to cardiac involvement, hemodynamic assessment, echocardiography, and therapeutic management across shock states. Because this was a narrative rather than systematic review, no formal risk-of-bias assessment or quantitative evidence synthesis was performed.

## 2. Pathophysiology of Cardiac Involvement

### 2.1. Cardiogenic Shock: From Primary Pump Failure to Systemic Circulatory Dysfunction

Cardiogenic shock differs from the other shock states discussed in this review because cardiac dysfunction is the initiating event rather than a secondary consequence of systemic circulatory failure. It develops when the heart cannot generate sufficient forward flow to meet tissue metabolic demands, most commonly in the setting of acute myocardial infarction, acute or acute-on-chronic heart failure, myocarditis, severe arrhythmias, or acute mechanical complications [8,13,14,15]. Acute valvular or mechanical causes, including acute severe mitral regurgitation, ventricular septal rupture, and other acute structural lesions, may produce an abrupt fall in forward flow and require urgent definitive intervention [13,14,15]. Although these conditions differ considerably in etiology, they share a final pathway of reduced stroke volume and cardiac output, abnormal ventricular loading, and impaired systemic perfusion.

In left ventricular-dominant shock, reduced systolic performance is often accompanied by an increase in left ventricular filling pressures and a fall in systemic arterial pressure. This combination is particularly unfavorable for the myocardium itself. As aortic diastolic pressure decreases and ventricular end-diastolic pressure rises, the effective coronary perfusion gradient narrows, worsening myocardial ischemia and further reducing contractility [14,16,17]. This creates a self-perpetuating cycle in which myocardial dysfunction causes hypotension, hypotension impairs coronary perfusion, and secondary ischemia further depresses cardiac output. The mechanism is most evident in acute myocardial infarction but may also occur in non-ischemic cardiogenic shock when low perfusion pressure, increased wall stress, and high myocardial oxygen demand coexist.

Compensatory neurohumoral responses initially support the circulation but may become maladaptive. Sympathetic activation increases heart rate (HR), contractility, and vascular tone, while activation of the renin–angiotensin–aldosterone system promotes vasoconstriction and fluid retention [14,15,17]. These responses can transiently maintain arterial pressure, but the resulting increase in systemic vascular resistance raises ventricular afterload and may further reduce stroke volume. Tachycardia also increases myocardial oxygen consumption while shortening diastolic coronary perfusion time. At the same time, venoconstriction and fluid retention increase ventricular filling pressures, favoring pulmonary and systemic congestion.

Right ventricular involvement adds further complexity. RV failure may occur because of right ventricular infarction, increased pulmonary vascular load, pre-existing pulmonary hypertension, or progression to biventricular dysfunction [13,14,15]. As RV output falls, LV preload also decreases. RV dilatation may further impair LV filling through septal displacement and ventricular interdependence, while systemic venous congestion contributes to renal and hepatic dysfunction. Cardiogenic shock should therefore not be considered solely a disorder of left ventricular contractility; LV-dominant, RV-dominant, and biventricular phenotypes can all produce inadequate systemic flow through different hemodynamic mechanisms.

The traditional description of cardiogenic shock as a uniformly low-output, vasoconstricted state is also incomplete. Severe myocardial injury and tissue hypoperfusion may trigger systemic inflammation, oxidative stress, endothelial dysfunction, and altered nitric oxide signaling [15,16,18]. In some patients, this inflammatory response promotes pathological vasodilation, producing a mixed cardiogenic–vasodilatory phenotype despite persistent myocardial dysfunction. The hemodynamic consequences of cardiogenic shock also depend on the interaction between ventricular contractility and arterial load. Loss of ventricular–arterial coupling may increase the energetic cost of ejection and further compromise effective forward flow, while cardiac power integrates pressure generation and flow as a global measure of pump performance [14,15,19]. Together with systemic and pulmonary congestion, these variables help characterize the severity of circulatory failure, although they should be interpreted as physiologic and prognostic descriptors rather than isolated therapeutic targets. This expanded model, initially proposed in myocardial infarction-related cardiogenic shock, helps explain why restoration of arterial pressure alone does not always restore adequate tissue perfusion [16]. More recent work similarly describes cardiogenic shock as a multisystem syndrome in which cardiac failure, abnormal vascular tone, inflammation, microcirculatory dysfunction, and organ injury interact [14,15,19].

These mechanisms ultimately extend the consequences of the initial cardiac insult well beyond the heart. Reduced cardiac output limits systemic oxygen delivery, whereas elevated venous pressures reduce the effective perfusion gradient across congested organs. Renal dysfunction, hepatic injury, metabolic acidosis, and increasing lactate may therefore result from the combined effects of low forward flow and venous congestion. This is particularly relevant in acute-on-chronic heart failure, where pre-existing congestion and neurohumoral activation are already present before the acute deterioration [19]. The heterogeneity of the syndrome is further supported by contemporary descriptions of non-congested, cardiorenal, and cardiometabolic phenotypes with different clinical trajectories and outcomes [20].

From the perspective of the present review, cardiogenic shock illustrates how a primary cardiac insult can evolve into the same downstream disturbances encountered in other forms of shock: altered preload and afterload, ventricular interdependence, impaired effective forward flow, vascular dysfunction, and tissue hypoperfusion. Echocardiography is therefore useful not only for documenting reduced ejection fraction, but for identifying the predominant ventricular phenotype, regional or global dysfunction, acute structural complications, RV involvement, loading conditions, and the adequacy of forward flow [15,21,22]. Serial assessment is especially important because the hemodynamic profile may evolve considerably during the course of shock and in response to treatment.

### 2.2. Septic Myocardial Depression

Myocardial dysfunction occurring during sepsis arises from a complex interaction of inflammatory signaling, metabolic alterations, and neurohumoral dysregulation that collectively impair cardiac performance. Although septic shock was historically thought to produce myocardial depression through global myocardial ischemia, studies have demonstrated preserved or even increased coronary blood flow in septic patients with ventricular dysfunction, indicating that impaired perfusion alone does not explain the observed cardiac abnormalities [12,23,24]. Current evidence instead suggests that myocardial depression is driven by a multifactorial process involving immune activation, intracellular metabolic disturbances, and systemic hemodynamic changes [25,26,27,28].

A key driver of septic myocardial depression is the intense inflammatory response triggered by infection. Pathogen-associated molecular patterns and damage-associated molecular patterns activate innate immune receptors such as toll-like receptors, leading to nuclear factor-κB signaling and the production of pro-inflammatory cytokines including tumor necrosis factor-α, interleukin-1, and interleukin-6 [12,29,30,31]. These mediators exert direct cardiodepressant effects and contribute to endothelial dysfunction, oxidative stress, and microvascular abnormalities that impair myocardial performance [23,25,26,27]. Additional inflammatory pathways involving mitogen-activated protein kinase (MAPK) signaling and immune cell activation have also been implicated in promoting cytokine release and myocardial injury in experimental models of septic cardiomyopathy [32].

Nitric oxide (NO) overproduction represents another important contributor to septic myocardial depression. Cytokine stimulation induces the expression of inducible NO synthase, resulting in sustained NO release that interferes with adrenergic signaling and reduces myocardial responsiveness to catecholamines [33,34,35]. NO-mediated activation of cyclic guanosine monophosphate pathways can further impair calcium sensitivity of contractile proteins and alter ventricular loading conditions, ultimately contributing to reduced myocardial contractility [12,36].

Disruption of cardiomyocyte calcium handling is also central to the pathogenesis of septic cardiomyopathy. Inflammatory mediators can suppress sarcoplasmic reticulum calcium transport proteins and modify the activity of L-type calcium channels and ryanodine receptors, impairing both systolic contraction and diastolic relaxation [37,38,39,40,41,42]. These abnormalities in intracellular calcium cycling may reduce myocardial contractility despite normal or elevated intracellular calcium concentrations, highlighting the complexity of cellular dysfunction during sepsis [23,43,44].

Metabolic disturbances within cardiomyocytes further contribute to myocardial dysfunction. Mitochondrial structural abnormalities and impaired oxidative phosphorylation reduce ATP generation and increase the production of reactive oxygen species, thereby limiting the energy available for cardiac contraction [12,45,46,47]. Experimental studies have also demonstrated disruption of mitochondrial dynamics, including abnormal fission, fusion, and impaired mitochondrial biogenesis, which further compromise cardiomyocyte metabolism and survival during septic states [32]. Oxidative stress associated with these processes can damage cellular membranes and contractile proteins, amplifying myocardial injury.

Beyond intrinsic cellular injury, systemic hemodynamic alterations during septic shock may exacerbate myocardial dysfunction. Profound vasodilation increases capillary permeability, and reduced vascular tone alters preload and afterload conditions and can negatively affect ventricular performance [23,24,25]. In addition, prolonged exposure to high concentrations of endogenous or exogenous catecholamines may induce structural myocardial injury, including contraction band necrosis and interstitial edema [12,48]. These factors highlight the interplay between systemic circulatory disturbances and intrinsic myocardial mechanisms in septic cardiomyopathy.

Myocardial edema and metabolic adaptation may further contribute to the transient cardiac phenotype of sepsis. Increased microvascular permeability and impaired lymphatic drainage can increase myocardial water content, thereby increasing ventricular stiffness and impairing contractility [12,49,50]. Despite multiple potentially injurious pathways, extensive irreversible cardiomyocyte death appears uncommon in human sepsis, supporting the concept that much of the myocardial dysfunction is functional and potentially reversible [51,52,53]. Metabolic “hibernation,” characterized by a temporary reduction in contractile activity to conserve cellular energy during severe systemic stress, has also been proposed as an adaptive mechanism contributing to this reversibility [45,54,55].

At the hemodynamic level, these interacting mechanisms translate into a highly dynamic and load-dependent cardiac phenotype characterized by variable combinations of systolic and/or diastolic dysfunction affecting both ventricles, often in the setting of profound vasoplegia and altered preload conditions [56]. Importantly, left ventricular ejection fraction may be preserved or even appear supranormal despite impaired intrinsic contractility, as reduced systemic vascular resistance lowers afterload and masks myocardial dysfunction [12]. Conversely, restoration of vascular tone may unmask underlying systolic impairment, highlighting the critical influence of loading conditions on cardiac assessment [12]. In addition, ventricular dilation—particularly of the left ventricle—may occur with relatively normal or low filling pressures, reflecting increased ventricular compliance rather than classic congestion [23]. The resulting phenotype is therefore heterogeneous, ranging from hyperdynamic circulation driven by vasoplegia to low-output states associated with septic cardiomyopathy, often evolving over time and in response to therapy [23]. Collectively, these features underscore that septic myocardial dysfunction is not a fixed entity but a dynamic interaction between intrinsic myocardial impairment and systemic hemodynamic alterations (Figure 1).

### 2.3. Hypovolemia and Cardiac Underfilling

#### 2.3.1. Hypovolemia as a Loading Dependent Cardiac Phenotype

While hypovolemic shock stems from intravascular volume loss rather than an initial myocardial insult, its early cardiac consequences primarily reflect altered loading conditions and critically reduced filling rather than intrinsic myocardial dysfunction [57]. As shock progresses, escalating autonomic activation, hypotension, and impaired oxygen delivery may shift the myocardium from adaptive compensation toward oxygen supply–demand mismatch and, in selected settings, secondary myocardial injury or cardiac dysfunction [58].

#### 2.3.2. Preload Collapse and the Left Ventricular Phenotype

The primary cardiac consequence of hypovolemia is a decline in venous return and central blood volume, resulting in reduced biventricular preload [58]. As end-diastolic filling decreases, stroke volume (SV) falls along the Frank–Starling relationship despite preserved intrinsic myocardial contractility [58]. Echocardiographically, severe preload depletion may manifest as reduced left ventricular end-diastolic dimensions, a small hyperdynamic cavity, and, in extreme cases, end-systolic cavity near obliteration [59,60]. Importantly, preserved or exaggerated systolic shortening may coexist with inadequate forward flow, reflecting ventricular underfilling rather than intrinsic systolic failure [61].

#### 2.3.3. Compensatory Sympathetic Activation and Adaptive Tachycardia

Once underfilling is established, baroreflex-mediated sympathetic activation and vagal withdrawal become central to maintaining circulatory stability [62]. The fall in SV is partly compensated by reflex tachycardia to preserve CO, while peripheral vasoconstriction supports arterial pressure and organ perfusion [62]. This compensatory pattern is especially important early in hypovolemia, when arterial pressure may remain relatively preserved despite substantial volume loss [63]. However, the hemodynamic benefit of adaptive tachycardia becomes progressively limited as shock advances [62].

#### 2.3.4. When Compensation Becomes Costly

As shock progresses, the compensatory mechanisms become increasingly costly. Persistent tachycardia shortens diastole, thereby limiting ventricular filling and coronary perfusion while increasing myocardial oxygen demand [64,65]. Although sympathetic vasoconstriction may initially preserve diastolic arterial pressure, progressive volume loss eventually lowers coronary perfusion pressure and reserve. This imbalance may be further aggravated when hemorrhage or anemia compromise systemic oxygen transport. Together, these changes progressively shift the myocardium toward oxygen supply–demand mismatch and ischemic stress, particularly within the subendocardium, where perfusion is especially vulnerable to shortened diastole and falling perfusion pressure [64].

#### 2.3.5. From Myocardial Stress to Acute Myocardial Injury

Once myocardial adaptive reserve is overwhelmed, severe or prolonged hypovolemic shock may progress beyond isolated preload failure and precipitate acute myocardial injury. Profound hypotension may reduce myocardial oxygen supply and generate a supply–demand imbalance. Under the 2026 Fifth Universal Definition of Myocardial Infarction, acute myocardial injury occurring in this setting should be classified as secondary myocardial infarction when accompanied by evidence of acute myocardial ischaemia and the relevant diagnostic criteria are fulfilled [66]. Under the previous Fourth Universal Definition, this mechanism would have been categorized as type 2 myocardial infarction [67]; earlier studies using that nomenclature identified anemia or bleeding, tachyarrhythmia, and hypotension among common provoking conditions [68]. Thus, severe or prolonged hypovolemia may create conditions for secondary myocardial injury and, when evidence of acute ischaemia is present, secondary myocardial infarction.

#### 2.3.6. Secondary Cardiac Involvement in Severe Hypovolemia

In selected patients, the hemodynamic consequences of hypovolemia may extend beyond isolated preload failure to secondary cardiac involvement. Marked ventricular underfilling and intense sympathetic activation may, in selected and likely uncommon cases, favor dynamic intraventricular or left ventricular outflow tract obstruction (LVOTO), particularly when hypovolemia coexists with predisposing conditions such as Takotsubo syndrome, acute myocardial infarction, or a structurally susceptible small hyperdynamic ventricle [69,70]. In this setting, the resulting fall in forward flow is better understood as a superimposed dynamic obstructive component rather than as primary cardiogenic pump failure [69]. In parallel, prolonged hemorrhagic or trauma-related shock may be complicated by secondary myocardial dysfunction as inflammatory, metabolic, and mitochondrial disturbances become superimposed on the initial deficit in ventricular filling [58].

### 2.4. Obstructive Shock: Mechanical Constraint and Ventricular Underfilling

Obstructive shock is a subtype of acute circulatory failure in which tissue hypoperfusion results from mechanical obstruction to cardiac filling, ventricular ejection, or central blood flow, rather than from primary myocardial contractile failure [2]. Although its hemodynamic presentation may overlap with cardiogenic shock, reduced forward flow in obstructive shock primarily reflects a mechanical impediment that alters ventricular loading or ejection rather than intrinsic myocardial dysfunction [2]. In clinical practice, the major causes of obstructive shock include acute pulmonary embolism (PE), cardiac tamponade, and tension pneumothorax [71].

#### 2.4.1. Right Ventricular Pressure Overload (Increased RV Afterload)

High-risk acute PE, particularly when accompanied by hemodynamic instability, provides the clearest model of obstructive shock driven by abrupt right ventricular afterload mismatch. Hemodynamic stability in this setting depends on effective right ventricle–pulmonary artery (RV–PA) coupling, namely the ability of right ventricular contractility to adapt to the impedance of the pulmonary circulation [72]. Because the right ventricle (RV) is physiologically designed for a low-pressure system, its tolerance for acute increases in afterload is limited. Once this adaptive reserve is exceeded, compensatory increases in contractility can no longer preserve SV, and RV–PA uncoupling develops [73,74]. The RV then dilates, wall stress rises, and systolic efficiency declines, shifting the chamber into a mechanically unfavorable pressure–length relationship [73,74]. The resulting hemodynamic disturbance extends beyond the RV. As right ventricular forward flow falls, pulmonary venous return and left ventricular (LV) preload decline, while septal shift and pericardial restraint further impair left ventricular filling through ventricular interdependence [75,76]. In severe PE, this may produce a coupled phenotype of RV strain and secondary LV underfilling, culminating in reduced systemic output and, in advanced cases, in overt circulatory collapse [72,73,76]. The clinical relevance of this RV–LV interaction is underscored by the prognostic value of the combined assessment of pulmonary artery pressure and left ventricular stroke volume (SV) in acute pulmonary embolism. These findings suggest that adverse outcomes are driven not simply by elevated RV afterload in isolation, but by the failure of the LV to sustain output as RV strain progresses [77].

#### 2.4.2. Impaired Cardiac Filling Due to External Constraint

Shifting the focus from the pulmonary circuit to the extravascular environment, cardiac tamponade introduces a second distinct mechanism of obstructive shock, in which rising intrapericardial pressure mechanically limits diastolic chamber expansion and progressively impairs ventricular filling and stroke volume generation [78,79]. Hemodynamic compromise emerges once intrapericardial pressure approaches and ultimately exceeds intracardiac filling pressures, thereby uncoupling venous return from effective ventricular filling and SV generation [79]. Because the thinner-walled right atrium and RV are particularly susceptible to external compression, right-sided chamber collapse and impaired diastolic filling often occur early, with a consequent fall in CO [78,79]. Under these conditions, ventricular interdependence becomes central to the physiology of shock, as the two ventricles compete for space within a constrained pericardial sac. During inspiration, enhanced right-sided filling occurs at the expense of left ventricular expansion, while septal shift further restricts left ventricular preload [75]. The resulting low-output state therefore reflects impaired biventricular filling and ventricular interdependence rather than primary myocardial pump failure [75,78,79]. In emergency department patients with moderate-sized or larger pericardial effusions, an internally validated four-variable prediction score (systolic blood pressure < 100 mmHg, effusion diameter, right ventricular diastolic collapse, and respiratory variation in mitral inflow velocity > 25%) stratified the 24 h risk of urgent pericardial drainage or tamponade-related death, illustrating the clinical relevance of combined hemodynamic and echocardiographic assessment [80,81].

#### 2.4.3. Reduced Venous Return from Elevated Intrathoracic Pressure

Tension pneumothorax represents a distinct form of obstructive shock in which pathological elevation of intrathoracic pressure impairs systemic venous return. As intrapleural pressure rises and becomes persistently positive, the gradient driving systemic venous return toward the right atrium falls, and right ventricular filling progressively declines [82,83]. In more advanced states, mediastinal displacement and compression of the cavoatrial junction and great veins further accentuate the reduction in preload [83]. The resulting reduction in RV output decreases pulmonary venous return and LV filling, producing a low-output state driven primarily by serial biventricular underfilling rather than intrinsic myocardial failure [82,83]. The hemodynamic consequence is therefore a sharp decline in effective CO driven primarily by impaired venous return and serial underfilling of both ventricles [82,83]. This physiology becomes even more precarious under positive-pressure ventilation, where additional increases in intrathoracic pressure may further reduce venous return and worsen RV loading conditions [82,84].

#### 2.4.4. Intrinsic Mechanical Obstruction to Intracardiac Flow

Less commonly, obstructive shock may arise from direct intracardiac obstruction to forward flow. This occurs in selected patients with structural lesions such as cardiac masses—most notably atrial myxomas causing intermittent valvular inflow obstruction—or in hypertrophic cardiomyopathy with severe LVOTO [85,86,87]. In contrast to the more prevalent forms of obstructive shock, where hemodynamic collapse is mediated by pulmonary vascular loading, pericardial restraint, or impaired venous return, these entities compromise circulation by directly obstructing intracardiac transit and limiting effective SV [69,85,86]. Although rare, these conditions should be recognized as mechanical causes of circulatory failure rather than myocardial pump dysfunction [69,86].

#### 2.4.5. Unifying Hemodynamic Consequences in Obstructive Shock

Despite their distinct initiating mechanisms, the major forms of obstructive shock converge on reduced effective forward flow through disruption of ventricular loading conditions [2,88]. Acute RV afterload excess, external cardiac constraint, impaired venous return, or direct intracardiac obstruction can impair pulmonary transit or ventricular filling, thereby reducing left ventricular preload and systemic stroke volume [75,76]. Ventricular interdependence further amplifies this process, particularly when RV dilation or pericardial constraint limits LV filling [75,76].

As obstructive shock progresses, however, secondary myocardial and systemic consequences may become superimposed on the initial mechanical disturbance. This transition is most evident in pressure-overload states, where rising right ventricular wall tension together with falling systemic arterial pressure progressively compromises right coronary perfusion, promotes ischemia of the already strained RV, and further depresses systolic performance [74,89]. Although this ischemic spiral is most clearly defined in acute right ventricular afterload excess, similar downstream deterioration may develop across obstructive states once CO and arterial pressure fall, as reduced forward flow limits tissue oxygen delivery and persistent low output and venous congestion promote lactate accumulation and cellular dysoxia [90,91]. Thus, the severity of obstructive shock increasingly reflects both the initiating mechanical lesion and the extent of secondary ischemic and metabolic amplification [88,89,90].

Recognizing these shared mechanisms is clinically important because obstructive shock may remain reversible before secondary myocardial and systemic consequences become established. Across the spectrum of obstructive shock, the most informative hemodynamic assessment is one that captures the interaction between pressure, flow, and biventricular performance rather than relying on any single variable in isolation [92,93]. Right ventricular strain, impaired ventricular filling, and declining systemic output should therefore be interpreted as components of an evolving hemodynamic process rather than as isolated findings. Early recognition of this trajectory may facilitate correction of the underlying mechanical cause before secondary ventricular dysfunction and systemic hypoperfusion become established [92,93].

### 2.5. Anaphylaxis: Vasodilation, Coronary Involvement, and Stress Cardiomyopathy Overlap

Anaphylactic shock is primarily characterized by profound vasodilation and intravascular volume loss related to increased vascular permeability. However, myocardial and coronary involvement may contribute in selected patients, producing a mixed hemodynamic phenotype [94,95].

#### 2.5.1. The Distributive Pathway and Loss of Vascular Resistance

The distributive component of anaphylactic shock is driven by a profound loss of peripheral vascular resistance and persistent vasodilation, as well as rapid plasma extravasation.

This vasoplegic collapse is primarily driven by the excessive activation of endothelial NO synthase and the resulting surge in NO. NO diffuses into adjacent vascular smooth muscle cells and leads to a prolonged state of muscle relaxation [96,97]. While NO is the central vasodilator mediator, the distributive collapse is further amplified by the synergistic vasodilatory effects of histamine, bradykinin, and platelet-activating factor [97].

Beyond active vasodilation, the release of histamine and platelet-activating factor precipitates a critical intravascular volume shift through the disruption of endothelial integrity. These mediators act on G-protein-coupled receptors, triggering the RhoA/ROCK pathway and myosin light chain kinase [98]. The resulting contraction of endothelial cells disrupts tight junctions, leading to a “capillary leak” syndrome where up to 35% of intravascular volume can shift to the extravascular space within 10 min [99].

The combination of the massive vasodilation and the “capillary leak” syndrome reduces venous return, and subsequently cardiac preload. This is reflected in rapid reductions in SV of approximately 4.2% [100].

Paradoxically, the vascular response is remarkably heterogeneous [101]. While systemic vascular resistance typically decreases due to NO-mediated vasodilation, specific beds such as the pulmonary, coronary, and portal systems undergo paradoxically intense vasoconstriction [102,103,104].

#### 2.5.2. Primary Myocardial Depression and Pump Failure

Experimental data suggest that the decline in CO cannot be explained solely by intravascular volume loss or reduced preload and may also reflect primary myocardial depression [105]. In experimental models of acute anaphylaxis, CO can drop by up to 90% alongside abrupt increases in left ventricular end-diastolic pressure by 35%, indicating primary myocardial pump failure rather than simple volume loss [105,106]. Moreover, specific mediators released during the allergic cascade, such as platelet-activating factor and histamine, have a potent negative inotropic effect, severely depressing myocardial contractility, and directly interact with myocardial ionic channels, promoting arrhythmic events [107].

#### 2.5.3. Coronary Circulation and Kounis Syndrome

Cardiac compromise in anaphylaxis is further driven by an intense increase in coronary artery resistance and subsequently coronary ischemia, clinically recognized as Kounis syndrome (allergic acute coronary syndrome) [108]. This syndrome represents the intersection of allergic inflammation and coronary circulation.

The human heart is densely populated with mast cells, heavily concentrated between myocardial fibers and wrapping the adventitia of both large epicardial coronary arteries and small intramural vessels. Their degranulation causes ischemia through multiple pathways [108].

Histamine classically acts on H1 receptors in the coronary vasculature, triggering intense, localized spasms in normal or nearly normal arteries. Histamine, along with leukotrienes, thromboxane, chymase, tryptase, and platelet-activating factor, can induce intense coronary vasospasm (Kounis type I), destabilize atherosclerotic plaques (Kounis type II), or trigger acute thrombosis or restenosis within previously deployed coronary stents (Kounis type III) [109,110].

Moreover, cardiac mast cells uniquely secrete massive amounts of renin and chymase [109,111]. Chymase acts as a local angiotensin-converting enzyme and rapidly converts angiotensin I to angiotensin II, heavily driving localized arteriolar vasoconstriction, stimulating sympathetic nerves to release noradrenaline, and precipitating arrhythmias [101,109]. Chymase also actively degrades pericellular matrix components like fibronectin and collagen [101,112]. This weakens the fibrous cap of stable plaques, inducing plaque rupture and classic myocardial infarction [101,113].

#### 2.5.4. Stress Cardiomyopathy Overlap

Adding a critical layer of complexity is the overlap with stress-induced (Takotsubo) cardiomyopathy. During severe anaphylaxis, patients experience a massive surge of endogenous catecholamines as a compensatory mechanism [114,115]. This may be compounded by exogenous epinephrine, which can contribute to myocardial stress. However, in a subset of patients, particularly those with concurrent Kounis syndrome or severe mast-cell activation, this catecholamine storm can paradoxically worsen myocardial ischemia and precipitate stress- induced cardiomyopathy (SICM) [116]. The transient left ventricular apical or basal ballooning further depresses global CO, complicating the allergic response with acute stress cardiomyopathy.

Therefore, recognizing the cardiogenic and stress-cardiomyopathy overlap in anaphylaxis is vital for diagnostic mapping. Advanced imaging confirms this primary cardiac involvement; for example, speckle-tracking echocardiography demonstrates significantly reduced global longitudinal strain (GLS) during acute anaphylaxis, reflecting acute myocardial dysfunction that resolves upon recovery [117].

## 3. Visualizing Cardiac Pathology with Echocardiography

As a form of circulatory failure, every state of shock, regardless of its etiology or type, implies an absolute or relative inability of the heart to reach adequate circulating support [2]. The wide availability, ease of use, and repeatability of echocardiography provide real-time, intermittent monitoring and assessment of hemodynamic status and cardiac performance [93,118]. Furthermore, temporal changes and responses to physiological or therapeutic challenges provide important insights that assist in diagnosis and guide management strategies [93].

Shock etiology determines the management, and several reports have incorporated echocardiography as a non-invasive and reliable tool to differentiate between different types [119]. Estimation of the cardiac index is the initial step in differentiating distributive shock from other types of shock. In cases of low cardiac index, inferior vena cava collapsibility and evidence of intravascular volume depletion suggest hypovolemic shock. Severely impaired left ventricular function, and in some cases right ventricular dysfunction, point toward cardiogenic shock. Finally, findings consistent with obstructive etiologies (i.e., pneumothorax, cardiac tamponade, or pulmonary embolism) indicate obstructive shock [2,120].

Beyond classification of shock etiology, echocardiography gives further insights into how different systemic conditions converge on cardiac dysfunction and may characterize the pattern of cardiac dysfunction (i.e., regional or global), the severity and the reversibility (i.e., evidence of viable or non-viable myocardium) [121].

Importantly, in shock states, the critical determinant is the ability of the heart to sustain adequate circulatory perfusion; thus, cardiac function should also be evaluated taking into consideration CO and SV [122].

### 3.1. Global and Segmental Dysfunction

When systolic performance is not intact, differentiation between global and segmental dysfunction should be part of echocardiographic evaluation.

Global hypokinesia should raise suspicion for myocardial depression due to diffuse inflammatory and metabolic impairment, as observed in sepsis [12]. However, this remains to be confirmed after excluding (Table 1) other potential diagnoses (i.e., like severe acidemia, history of heart failure, acute myocarditis etc.) [123,124]. Extensive coronary artery disease, particularly three-vessel disease, may also be accompanied by chronic global hypokinesia, especially in the setting of supply–demand mismatch (Table 1).

Segmental wall-motion abnormalities should prompt evaluation for alternative or coexisting etiologies, including acute ischemia, prior infarction, stress-induced cardiomyopathy, and myocarditis; however, regional abnormalities do not by themselves exclude sepsis-related myocardial dysfunction (Table 2). Newly identified hypokinesia corresponding to a coronary territory should raise suspicion for myocardial ischemia or injury and should be interpreted together with the clinical presentation, ECG findings, and cardiac biomarkers. Evidence such as elevated cardiac biomarkers, ECG abnormalities, and newly developed regional wall motion abnormalities support the diagnosis and underline the importance of serial echocardiographic evaluation with appropriate image archiving and comprehensive documentation of findings. Additionally, echogenic and thinned myocardial segments, particularly when corresponding to ECG findings of prior myocardial infarction, suggest chronic cardiomyopathy of ischemic origin.

SICM may present with similar findings, making the differential diagnosis challenging. Discrepancy between the extent of wall motion abnormalities and the degree of elevation in cardiac biomarkers or ECG changes, as well as the temporal evolution of findings, may aid in differentiation [125]. Evidence of physical or emotional stress is supportive of the diagnosis. However, in critically ill patients, the presence of physical stress cannot reliably differentiate between left ventricular dysfunction due to stress-induced cardiomyopathy and that of ischemic origin.

When myocarditis is the cause of regional wall motion abnormalities, these are more commonly observed in the inferior and posterior walls and less frequently in the septum. Myocarditis may also be associated with increased wall thickness due to myocardial edema and can coexist with pericarditis [126,127]. However, the clinical findings accompanying myocarditis, such as chest pain, are not present in critically ill patients in shock (Table 2).

### 3.2. Cardiac Output and Left Ventricular Outflow Tract Velocity–Time Integral (LVOT VTI)

Understanding the adequacy of cardiac function in hemodynamically unstable patients extends far beyond the fraction of left ventricular end-diastolic volume ejected during systole. Accordingly, in critically ill patients, the CO, and more precisely cardiac index (CI), should be considered when evaluating left ventricular systolic performance. Echocardiographically, LVOT VTI can be used either to calculate CO or as a surrogate of CO to assess forward flow and cardiac performance, even when left ventricular ejection fraction (LVEF) appears normal, identifying low-flow states [128]. When mean arterial pressure (MAP) and right atrial pressure (RAP) are available, echocardiographically derived CO can also contribute to estimation of systemic vascular resistance (SVR) according to the relationship SVR = 80 × (MAP − RAP)/CO. This may provide additional physiological context when distinguishing vasoplegic low-resistance states from shock phenotypes characterized by increased systemic vascular tone [129]. An LVOT VTI of approximately 15 cm or less has been associated with low-flow states and adverse prognosis in selected cohorts, but should not be interpreted as a standalone diagnostic or treatment threshold [130]. To the contrary, higher LVOT VTI values, especially when accompanied by low systemic vascular resistance, may be attributed to septic cardiomyopathy [131].

### 3.3. Deformation Imaging

Assessment of myocardial deformation through strain analysis provides additional insight into the impact of different types of shock on myocardial performance. GLS is the most extensively studied parameter and represents a sensitive marker directly associated with left ventricular systolic function [132]. Segmental assessment of left ventricular strain may identify regional pathology not yet evident on visually estimated systolic function or wall motion score index [132]. Accordingly, in myocarditis or septic cardiomyopathy, left ventricular systolic dysfunction may be present despite a normal ejection fraction, particularly in the setting of reduced afterload, and may be detected early by decreased global or regional longitudinal strain values [133]. However, strain analysis is technically demanding and requires more comprehensive image acquisition and analysis, which may limit its routine use in critically ill patients; moreover, findings should be interpreted within the broader clinical context because septic cardiomyopathy remains a diagnosis that requires exclusion of alternative causes of myocardial dysfunction [132].

### 3.4. Assessment of Diastolic Function

Diastolic function assessment is an integral part of every echocardiographic assessment. Nevertheless, in critically ill patients, tachycardia and arrhythmias can impair the accuracy and reliability of diastolic indices, limiting their clinical applicability. Despite these limitations the ratio of the transmitral early E wave to atrial contraction A wave (E/A ratio) and the ratio of early transmitral E wave to tissue e prime wave (E/e’ ratio) should be recorded and interpreted cautiously [134]. Additional data may be provided by the measurement of left atrial strain, which can also be used to predict responsiveness to volume infusion [135,136]. Accordingly, in the presence of elevated filling pressures or a restrictive filling pattern, low CO should not be managed with aggressive fluid resuscitation. Instead, these findings should prompt consideration for diagnoses, including ischemic cardiomyopathy, septic cardiomyopathy, advanced heart failure, or restrictive cardiomyopathies, each associated with distinct clinical phenotypes and management strategies [72].

### 3.5. Right Ventricular Assessment

Assessment of the RV provides critical insight into the underlying etiology of shock (Table 3). In pulmonary embolism obstructive shock, acute right ventricular (RV) dilatation and dysfunction help in diagnosis, offer prognostic information, and guide evaluation of treatment efficacy [72].

In states of increased pulmonary vascular resistance, pulsed-wave Doppler interrogation of the right ventricular outflow tract (RVOT) may provide additional information regarding RV afterload and RV–pulmonary arterial coupling. Increasing pulmonary vascular resistance is associated with shortening of RVOT acceleration time and alteration of RVOT velocity–time integral morphology from a normal parabolic contour toward a more triangular pattern, with midsystolic notching potentially occurring as pulmonary vascular impedance increases [137,138].

Mechanical ventilation, especially in ARDS patients with high positive pressures, may cause dysfunction of the RV, which, when significant, may also impair the preload of the left ventricle and systemic CO [139].

Septic cardiomyopathy may also impair right ventricular performance. However, isolated right ventricular dysfunction is relatively uncommon and, when present, is usually mild. Moreover, the RV is not dilated since the primary cause of dysfunction in this scenario is neither pressure nor volume overload [12].

Cardiac tamponade is a clinical diagnosis. However, echocardiographic findings can support the identification of impending tamponade, including the presence of pericardial effusion and respiratory variation in mitral inflow or left ventricular outflow tract (LVOT) forward flow. Right ventricular free wall collapse is a highly specific sign and explains the underlying cause of a low CO state [140] (Table 3).

### 3.6. Practical Early Integrated Echocardiography Approach in Undifferentiated Shock

Given the marked hemodynamic heterogeneity of shock and the limitations of isolated clinical or laboratory assessment, an “early integrated echocardiography” approach may provide a structured method for early shock phenotyping. In patients presenting with persistent hypoperfusion or hemodynamic instability, bedside echocardiography allows rapid assessment of cardiac function and circulatory status and may assist in identifying the dominant mechanism responsible for circulatory failure [12,141,142]. Rather than relying exclusively on the traditional etiologic classification of shock, echocardiographic assessment can therefore provide a complementary physiology-based characterization of the patient’s hemodynamic phenotype [12,23,59].

The initial examination should first address immediately reversible obstructive or structural causes of shock, including pericardial tamponade, massive pulmonary embolism, severe acute valvular abnormalities, or other major mechanical causes of circulatory compromise [12,23,59]. Once these conditions have been excluded, a systematic assessment should integrate left ventricular (LV) systolic function, right ventricular (RV) size and performance, forward flow, cardiac filling, preload responsiveness, pulmonary and systemic venous congestion, and fluid tolerance [23,142]. Importantly, LV ejection fraction should not be interpreted in isolation because of its marked load dependence; in profound vasoplegia, LVEF may remain preserved or even appear supranormal despite impaired intrinsic myocardial function [12,59].

From a practical bedside perspective, the echocardiographic examination should address a sequence of physiologically relevant questions: (1) Is an immediately reversible obstructive or structural cause present? (2) Is LV systolic function impaired, preserved, or hyperdynamic? (3) Is there RV dilatation, dysfunction, or evidence of increased RV afterload? (4) Is forward flow reduced, as assessed by LVOT VTI or calculated cardiac output? (5) Is there evidence of persistent preload dependence? and (6) Is pulmonary or systemic venous congestion present and therefore limiting further fluid administration? Evaluation of RV function is particularly important in mechanically ventilated patients, in whom increased pulmonary vascular resistance and heart–lung interactions may substantially modify RV loading conditions and performance [84,143].

Assessment of preload and fluid responsiveness should similarly rely on an integrated approach rather than on a single static parameter. Inferior vena cava dynamics, LVOT VTI, and dynamic maneuvers such as passive leg raising may provide complementary information regarding preload responsiveness, while lung ultrasound and assessment of venous congestion may help identify patients in whom additional fluid administration could be harmful [12,23,59]. Diastolic indices may further refine estimation of filling conditions in selected patients, although their interpretation should remain cautious in the presence of tachycardia, arrhythmias, mechanical ventilation, and rapidly changing loading conditions [12,23].

Based on this integrated assessment, patients may be characterized according to the predominant hemodynamic phenotype, including LV systolic dysfunction, hyperdynamic vasoplegia, RV failure, persistent hypovolemia, obstructive physiology, or mixed/indeterminate shock [12,23,84,144,145]. Importantly, such phenotypes should not be regarded as fixed categories, as different mechanisms may coexist and evolve during the course of critical illness and in response to therapy. The proposed early integrated echocardiography framework therefore provides an initial physiological framework for bedside classification while emphasizing the need for subsequent reassessment as the clinical and hemodynamic profile changes (Figure 2). Importantly, echocardiographic assessment should be performed concurrently with initial stabilization and etiologic treatment and must not delay time-critical interventions. Regional wall-motion abnormalities may support a diagnosis of acute myocardial ischemia, but a normal or nondiagnostic echocardiographic examination does not exclude acute coronary syndrome; clinical presentation, electrocardiography, cardiac biomarkers, and established reperfusion pathways remain essential.

The value of identifying these phenotypes lies ultimately in their therapeutic implications, because the same arterial pressure may require fundamentally different interventions depending on whether inadequate flow reflects preload deficiency, vasoplegia, ventricular failure, or mechanical obstruction.

## 4. Therapeutic Implications

The strength of evidence supporting therapeutic interventions differs substantially across shock phenotypes. Throughout this section, guideline-supported or trial-supported recommendations should be distinguished from strategies based predominantly on physiological rationale, observational evidence, or expert consensus. Established practices include treatment of the underlying etiology, norepinephrine as first-line vasopressor therapy in septic shock, dynamic assessment of fluid responsiveness, and prompt definitive treatment of reversible obstructive causes. In contrast, the use of individual echocardiographic parameters to select a specific vasoactive or inotropic agent, advanced measures of ventricular–arterial coupling, and ultrasound-guided de-resuscitation strategies remain less extensively validated and should be interpreted within the broader clinical and hemodynamic context.

### 4.1. Echocardiography-Guided Fluid Management in Hypovolemic and Distributive Shock

Early intravascular volume expansion remains an important component of resuscitation in hypovolemic and selected distributive shock states; however, excessive fluid administration may contribute to pulmonary edema, tissue congestion, and organ dysfunction. Because static preload indices such as central venous pressure have limited ability to predict fluid responsiveness, contemporary resuscitation increasingly favors dynamic and physiology-based assessment. Since only a subset of critically ill patients are fluid responsive, echocardiographic assessment of cardiac function, preload dependence, and dynamic changes in stroke volume can help individualize fluid administration and simultaneously assess fluid tolerance [146].

#### 4.1.1. Cardiac Ultrasound Assessment

The clinical benefit of ultrasound-guided resuscitation compared with conventional management remains incompletely established; however, point-of-care ultrasound (POCUS), incorporating focused cardiac ultrasound (FoCUS), provides rapid bedside assessment of cardiovascular physiology in undifferentiated hypotension and shock [147,148,149]. Integration of ventricular performance, forward flow, inferior vena cava (IVC) dynamics, and pulmonary findings can help characterize preload responsiveness, venous congestion, and fluid tolerance. Importantly, fluid responsiveness is neither universal nor static and should therefore be reassessed as the hemodynamic state evolves [146].

Changes in LVOT velocity–time integral (VTI) can be used as a surrogate for changes in stroke volume and cardiac output during dynamic preload assessment [149]. Passive leg raising (PLR) provides a reversible preload challenge by transiently mobilizing venous blood without administering exogenous fluid; an increase in LVOT VTI of approximately 10–15% following PLR has been used to identify fluid responsiveness [149]. Integration of PLR-induced VTI changes with other ultrasonographic findings may further improve physiological characterization in heterogeneous critically ill populations [150].

#### 4.1.2. Inferior Vena Cava and Dynamic Assessment

The IVC is frequently assessed as an adjunctive marker of preload dependence, but respiratory variation in IVC diameter should not be interpreted in isolation. Mechanical ventilation, spontaneous respiratory effort, intra-abdominal pressure, and RV dysfunction can substantially alter IVC dynamics and limit diagnostic specificity [147,151]. Accordingly, IVC assessment should be integrated with Doppler-derived changes in stroke volume, dynamic preload testing, and lung ultrasound rather than used as a standalone determinant of fluid administration [147,152].

Assessment of systemic venous congestion can be complemented by the Venous Excess Ultrasound Score (VExUS), which integrates IVC diameter with hepatic, portal, and renal venous Doppler patterns [153]. VExUS may help identify patients with clinically relevant venous congestion in whom further fluid administration could increase venous hypertension, although evidence for outcome benefit from VExUS-guided treatment remains limited [153].

#### 4.1.3. Lung Ultrasound and Fluid Tolerance

Prediction of fluid responsiveness alone is insufficient because a patient may remain preload responsive while being unable to tolerate additional volume. Lung ultrasound complements cardiac assessment by identifying interstitial fluid accumulation and pulmonary edema; increasing B-lines during resuscitation may indicate declining fluid tolerance before overt clinical congestion develops [146,151,152,153]. Integration of cardiac and lung ultrasound therefore allows preload responsiveness to be interpreted alongside the risk of fluid accumulation when considering further volume administration.

#### 4.1.4. Clinical Integration

Recent systematic reviews suggest that POCUS-guided resuscitation may reduce fluid administration and modify hemodynamic management, although heterogeneity among studies and uncertainty regarding patient-centered outcomes preclude treating these approaches as validated therapeutic algorithms [154]. POCUS is therefore best viewed as an integrative tool combining cardiac, pulmonary, and vascular assessment within the broader clinical context [155].

Despite its advantages, several limitations persist. The technique is highly operator-dependent and requires adequate training and experience to ensure accurate interpretation [148]. Image acquisition may be challenging in certain patient populations, particularly those with obesity or poor acoustic windows. Furthermore, echocardiography provides intermittent rather than continuous data, which may limit its responsiveness in rapidly changing clinical cases [147,156].

Complex hemodynamic states, such as combined septic and cardiogenic shock or significant right ventricular dysfunction, may further complicate interpretation [149]. For these reasons, echocardiography should be integrated with clinical assessment and other monitoring modalities to provide a comprehensive evaluation of the patient [150]. While recent studies are promising, heterogeneity in study design and patient populations means that further large-scale randomized trials are still needed to define optimal protocols and standardize practice.

### 4.2. Recognizing Need for Vasopressors vs. Inotropes

Selection of vasoactive support should be guided by the predominant hemodynamic abnormality rather than by shock classification alone. Hypotension may result predominantly from reduced systemic vascular resistance, inadequate cardiac output, or a combination of both, while RV dysfunction may further modify the response to therapy [157]. Accordingly, bedside assessment should assist the principal therapeutic priority: restoration of vascular tone, augmentation of cardiac output, correction of preload, or treatment of RV afterload [158,159].

#### 4.2.1. Vasoactive Agents

Choosing vasoactive agents based on the underlying shock mechanism may optimize hemodynamics and limit adverse effects.

Vasopressors increase MAP by augmenting vascular tone and include catecholamines (norepinephrine, epinephrine, dopamine) and non-catecholaminergic agents (e.g., vasopressin, phenylephrine, terlipressin, selepressin) [160]. Catecholamines exert dose-dependent effects, ranging from predominant inotropy and chronotropy at lower doses to vasoconstriction at higher doses [161]. Norepinephrine is the recommended first-line vasopressor in septic shock [155] and is commonly favored when vasopressor support is required in cardiogenic shock because of its hemodynamic profile and lower arrhythmogenic potential than dopamine [14,15,19,21,162]. Particularly in preload-responsive patients, norepinephrine may also increase venous return and augment CO through β1-mediated inotropic effects [163,164]. Vasopressin is commonly used as an adjunctive non-catecholaminergic vasopressor in septic or vasodilatory shock, whereas phenylephrine is generally reserved for selected situations in which β-adrenergic stimulation is undesirable or dynamic LV outflow-tract obstruction is present [155,157], while newer agents such as selepressin, angiotensin II, and terlipressin have not demonstrated clear mortality benefit over norepinephrine [165,166,167].

Inotropes (inodilators and inopressors) increase CO by enhancing myocardial contractility. Inodilators are used to improve tissue perfusion and reverse markers of hypoperfusion in low-CO states such as cardiogenic shock, but may also be considered in septic shock with persistent hypoperfusion despite adequate fluids and blood pressure, particularly in the presence of sepsis-related myocardial dysfunction [155,168,169]. Most commonly used agents are dobutamine and milrinone, with dose-dependent increases in contractility and HR. Phosphodiesterase-3 inhibitors such as milrinone may be considered in selected patients with low-output states, including selected cases with RV dysfunction or reduced β-adrenergic responsiveness, while recognizing their vasodilatory effects, prolonged half-life, and potential to worsen hypotension [157,170,171]. Levosimendan has not demonstrated a mortality benefit over dobutamine in septic shock and may be limited by vasodilatory adverse effects and its prolonged half-life [2,172]. In hypovolemic and obstructive shock, vasoactive agents are adjunctive to correction of the underlying cause. In hypovolemia, restoration of intravascular volume is fundamental, whereas in obstructive shock definitive targeted treatment takes priority; inotropes or vasopressors may be required temporarily when myocardial or RV dysfunction or severe hypotension persists [2,173,174]. Unresponsive hypotension despite vasopressors is associated with high mortality and may reflect vasoplegia, metabolic derangements, or receptor dysfunction [157,175,176,177,178]. Its management includes reassessment of cardiac filling, preload responsiveness, congestion, and fluid tolerance, addition of vasopressin [179,180], escalation to epinephrine, and consideration of hydrocortisone in refractory septic shock to reduce catecholamine requirements, although without proven mortality benefit [176,181,182].

#### 4.2.2. Therapeutic Targets

Vasopressors are titrated to maintain an arterial pressure compatible with adequate organ perfusion. In septic or predominantly vasodilatory shock, a MAP of approximately 65 mmHg is commonly used as an initial target, but the optimal pressure should be individualized according to the underlying shock mechanism, chronic blood-pressure status, and evidence of organ perfusion [155]. Inotropic therapy may be considered when persistent hypoperfusion is attributable to inadequate cardiac output despite adequate preload and perfusion pressure; treatment response should be assessed using integrated clinical and hemodynamic markers rather than arbitrary cardiac-output targets [155].

In clinical routine, use and titration of vasoactive agents is individualized and related to patients’ comorbidities, physiological characteristics, etiology of shock, and physicians’ experience with available vasoactive agents. The decision to initiate vasoactive support should be based on the underlying hemodynamic mechanism rather than on a broad etiologic grouping. In vasodilatory shock, persistent hypotension accompanied by evidence of tissue hypoperfusion may support vasopressor therapy after appropriate assessment of preload and reversible causes. Capillary refill time (CRT) and the central-to-toe temperature difference (Tc–toe), among others, have been proposed as objective parameters of peripheral perfusion [155]. Lactate and central venous oxygen saturation (ScvO_2_) are influenced by oxygen delivery, oxygen extraction, anemia, hypoxemia, metabolic stress, and other physiological factors and should therefore be interpreted in context rather than as organ-specific or standalone treatment targets [155]. Similarly, the hemodynamic effects of individual vasoactive agents are dose- and phenotype-dependent and should not be interpreted as fixed responses (Table 4).

#### 4.2.3. Echocardiography for the Dilemma Vasopressors vs. Inotropes

Echocardiography provides a rapid, non-invasive means of defining the predominant hemodynamic phenotype and supporting individualized vasoactive therapy in shock. Rather than relying on isolated parameters, integration of ventricular function, forward flow, filling conditions, and preload responsiveness can help distinguish vasoplegia from myocardial dysfunction or hypovolemia [4,141]. A low-flow state with impaired LV contractility may support inotropic therapy, whereas preserved or hyperdynamic flow in the presence of persistent hypotension favors vasopressor treatment. Dynamic changes in LVOT VTI may additionally help determine whether further fluid administration is likely to increase stroke volume [155]. Assessment of RV function and afterload is particularly important because significant RV dysfunction may require avoidance of excessive vasoconstriction and adoption of RV-directed strategies [4,141]. Echocardiographic findings should therefore be interpreted as components of an evolving physiological profile rather than as fixed treatment thresholds. Serial reassessment after therapeutic interventions allows adjustment of fluids, vasopressors, and inotropes according to the changing hemodynamic state [4,141] (Figure 3).

#### 4.2.4. Pulmonary Artery Catheterization (PAC) for the Dilemma: Vasopressors vs. Inotropes

Pulmonary artery catheterization (PAC) provides detailed invasive hemodynamic characterization and may be particularly useful in refractory or complex shock when the predominant mechanism remains uncertain despite initial assessment [14,183]. Simultaneous evaluation of cardiac output/index, filling pressures, SVR, pulmonary vascular resistance (PVR), and SvO_2_ can distinguish low-flow pump failure, vasoplegia, preload deficiency, congestion, and predominant RV dysfunction [184]. Low cardiac output with elevated filling pressures may support inotropic and decongestive strategies, whereas low SVR with preserved output favors vasopressor therapy. Conversely, low filling pressures may identify patients in whom cautious fluid administration remains appropriate [14,183]. Additional indices such as cardiac power output and PAPi can further characterize global pump performance and RV–pulmonary vascular interaction [14,183]. PAC-derived variables should be integrated with clinical and echocardiographic findings rather than interpreted as isolated therapeutic thresholds. Repeated hemodynamic assessment can subsequently guide adjustment of fluids, vasopressors, inotropes, and RV-directed therapy according to treatment response [14] (Figure 4).

## 5. Integration into Multidisciplinary Shock Management

### 5.1. How Cardiology Contributes to ICU and Emergency Settings

The management of shock has progressively evolved into a multidisciplinary process in which cardiology plays a central role in both diagnosis and treatment. Beyond identifying primary cardiogenic shock, cardiologists are essential in recognizing secondary myocardial dysfunction in systemic conditions such as sepsis, where cardiac involvement is often dynamic and load-dependent. Differentiating between primary cardiac failure and mixed shock states is critical, as it directly influences therapeutic strategy and outcomes [8,185].

In contemporary intensive care and emergency settings, cardiology contributes to detailed hemodynamic profiling and treatment optimization. Through the integration of bedside echocardiography, advanced hemodynamic monitoring, and interpretation of complex cardiovascular interactions, cardiologists guide decisions regarding fluid resuscitation, vasoactive agents, and inotropic support [8]. In refractory or complex cases, they are instrumental in identifying candidates for mechanical circulatory support (MCS), including venoarterial extracorporeal membrane oxygenation and percutaneous ventricular assist devices, particularly when a cardiogenic component coexists or emerges during the course of illness [14,15,19,21].

### 5.2. Serial Echocardiographic Reassessment and Multidisciplinary Integration

The clinical value of echocardiography in shock extends beyond the initial identification of the predominant hemodynamic phenotype. Shock represents a dynamic process in which ventricular function, preload, afterload, pulmonary vascular loading, and systemic vascular tone may change substantially following fluid administration, vasoactive or inotropic therapy, mechanical ventilation, progression of the underlying disease, or definitive treatment of its cause [12,23]. Accordingly, echocardiographic findings should not be interpreted as a single diagnostic snapshot. Serial reassessment after major therapeutic interventions allows evaluation of whether the intended physiological response has occurred and whether the initial phenotype has persisted, resolved, or evolved into a mixed circulatory state [84,141,145].

### 5.3. Controversies, Limitations of Current Evidence, and Future Directions

Despite increasing recognition of cardiac dysfunction across shock states, important uncertainties limit the interpretation of the available literature. This is particularly evident in sepsis-induced cardiomyopathy, for which no universally accepted diagnostic definition exists [56]. Reported prevalence varies substantially according to the timing of assessment, loading conditions, and the echocardiographic parameter used. Left ventricular ejection fraction is highly afterload dependent and may therefore underestimate intrinsic myocardial dysfunction during profound vasoplegia, whereas global longitudinal strain may detect subtler abnormalities but remains dependent on image quality, technical expertise, and methodological standardization [23]. Similarly, right ventricular dysfunction in sepsis cannot always be attributed to intrinsic septic myocardial injury because mechanical ventilation, hypoxemia, hypercapnia, pulmonary vascular loading, and concomitant pulmonary disease may independently impair RV performance. Consequently, a single echocardiographic abnormality should not be interpreted as diagnostic of septic cardiomyopathy without consideration of loading conditions, competing diagnoses, and serial evolution [56].

Similar limitations apply to echocardiography-guided management. Contemporary critical-care guidance supports critical care ultrasonography for hemodynamic assessment in septic and cardiogenic shock and suggests its use for targeted volume management [186]. Nevertheless, the evidence is considerably stronger for echocardiography as a diagnostic and physiological monitoring tool than for individual echo-derived thresholds or phenotype-to-treatment algorithms as outcome-proven therapeutic strategies. Parameters such as IVC respiratory variation, LVOT VTI, E/e′, VExUS, and indices of RV–pulmonary arterial coupling are influenced by ventilation, rhythm, loading conditions, acquisition technique, and the clinical context. They should therefore be integrated rather than applied as isolated therapeutic triggers [186].

Therapeutic evidence is also uneven across shock phenotypes. Some interventions, such as norepinephrine as first-line vasopressor therapy in septic shock and immediate correction of reversible mechanical causes of obstructive shock, are supported by guideline-level evidence. In contrast, the optimal timing of additional vasopressors, selection of inotropic support according to specific echocardiographic phenotypes, advanced ultrasound-guided de-resuscitation strategies, and several RV-directed interventions remain supported predominantly by physiological rationale, observational evidence, or expert consensus. Accordingly, Figure 2, Figure 3 and Figure 4 should be interpreted as conceptual, physiology-based frameworks intended to organize hemodynamic assessment and clinical reasoning rather than as validated treatment algorithms or outcome-proven management pathways. Future prospective studies should evaluate standardized definitions of cardiac dysfunction, serial rather than single-time-point echocardiographic phenotyping, and whether integrated phenotype-guided treatment strategies improve patient-centered outcomes compared with conventional hemodynamic management. The recent literature continues to emphasize both the need for individualized septic-shock management and uncertainty surrounding several adjunctive vasoactive strategies [187].

## 6. Conclusions

Shock states arise from distinct pathophysiological mechanisms but may converge on impaired effective forward flow, altered ventricular loading, myocardial dysfunction in selected settings, and tissue hypoperfusion. Echocardiography provides a rapid means of characterizing ventricular function, loading conditions, forward flow, and reversible mechanical causes, while serial reassessment can identify evolving or mixed hemodynamic phenotypes. Integration of echocardiographic findings with clinical assessment and, when required, invasive hemodynamic data may support individualized resuscitation. However, individual echocardiographic or PAC-derived parameters should not be interpreted as standalone therapeutic targets, and several proposed phenotype-guided treatment strategies remain dependent on physiological rationale and observational evidence rather than outcome-proven algorithms.

## Figures and Tables

**Figure 1:**
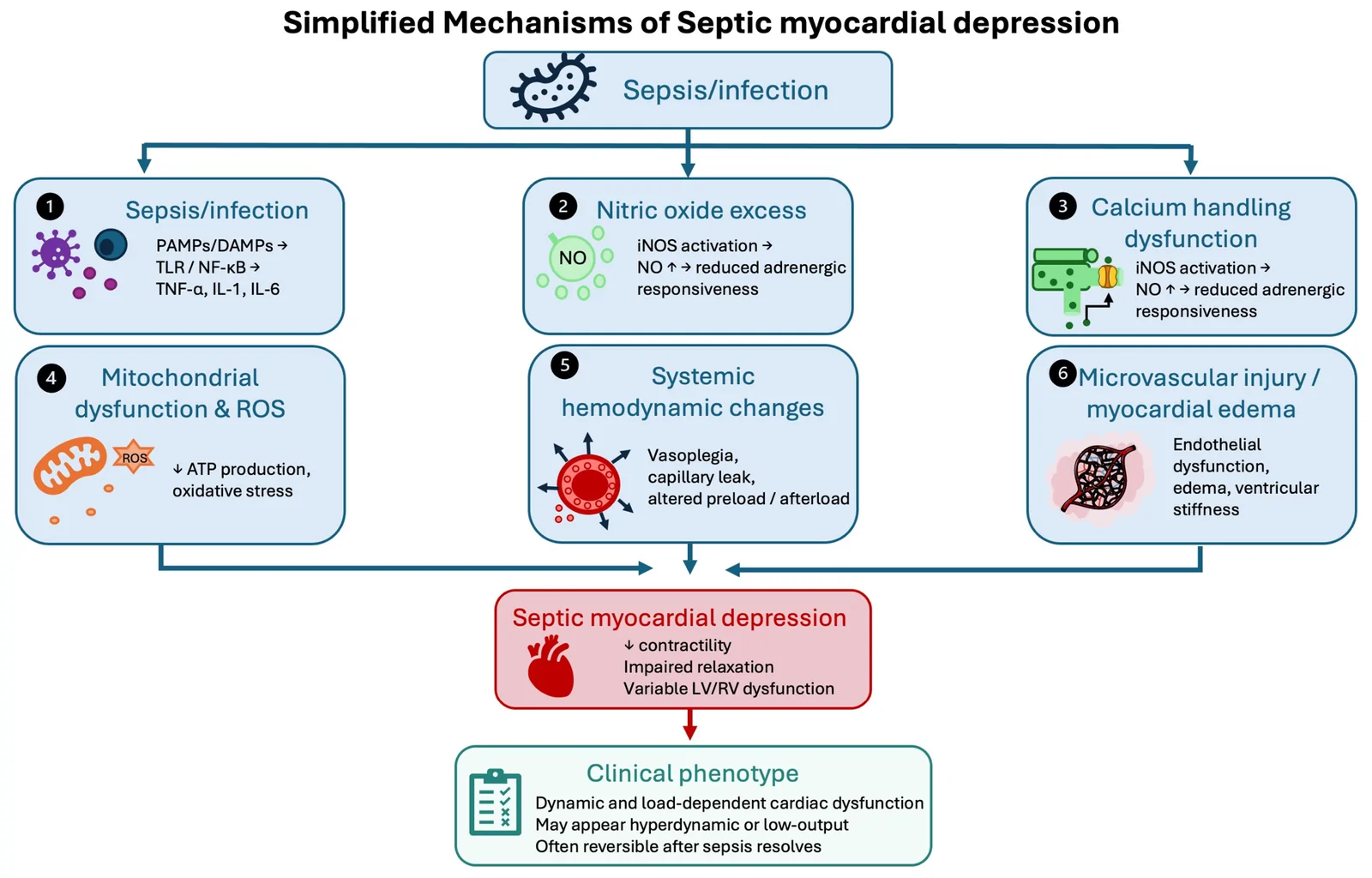
Simplified mechanisms of septic myocardial depression. Sepsis-induced myocardial dysfunction results from the interaction of multiple inflammatory, cellular, metabolic, and hemodynamic mechanisms rather than from global myocardial ischemia alone. Infection triggers innate immune activation through pathogen- and damage-associated molecular patterns, leading to cytokine release, nitric oxide overproduction, oxidative stress, mitochondrial dysfunction, impaired calcium handling, endothelial and microvascular injury, and myocardial edema. These processes collectively reduce myocardial contractility, impair relaxation, and contribute to variable left and right ventricular dysfunction. At the clinical level, the phenotype is dynamic and load-dependent, ranging from hyperdynamic circulation in the setting of vasoplegia to low-output states when intrinsic myocardial depression predominates. Importantly, septic myocardial depression is often reversible after control of infection and resolution of the systemic inflammatory response. (Image(s) provided by Servier Medical Art (https://smart.servier.com), licensed under CC BY 4.0 (https://creativecommons.org/licenses/by/4.0/, accessed on 20 April 2026).

**Figure 2:**
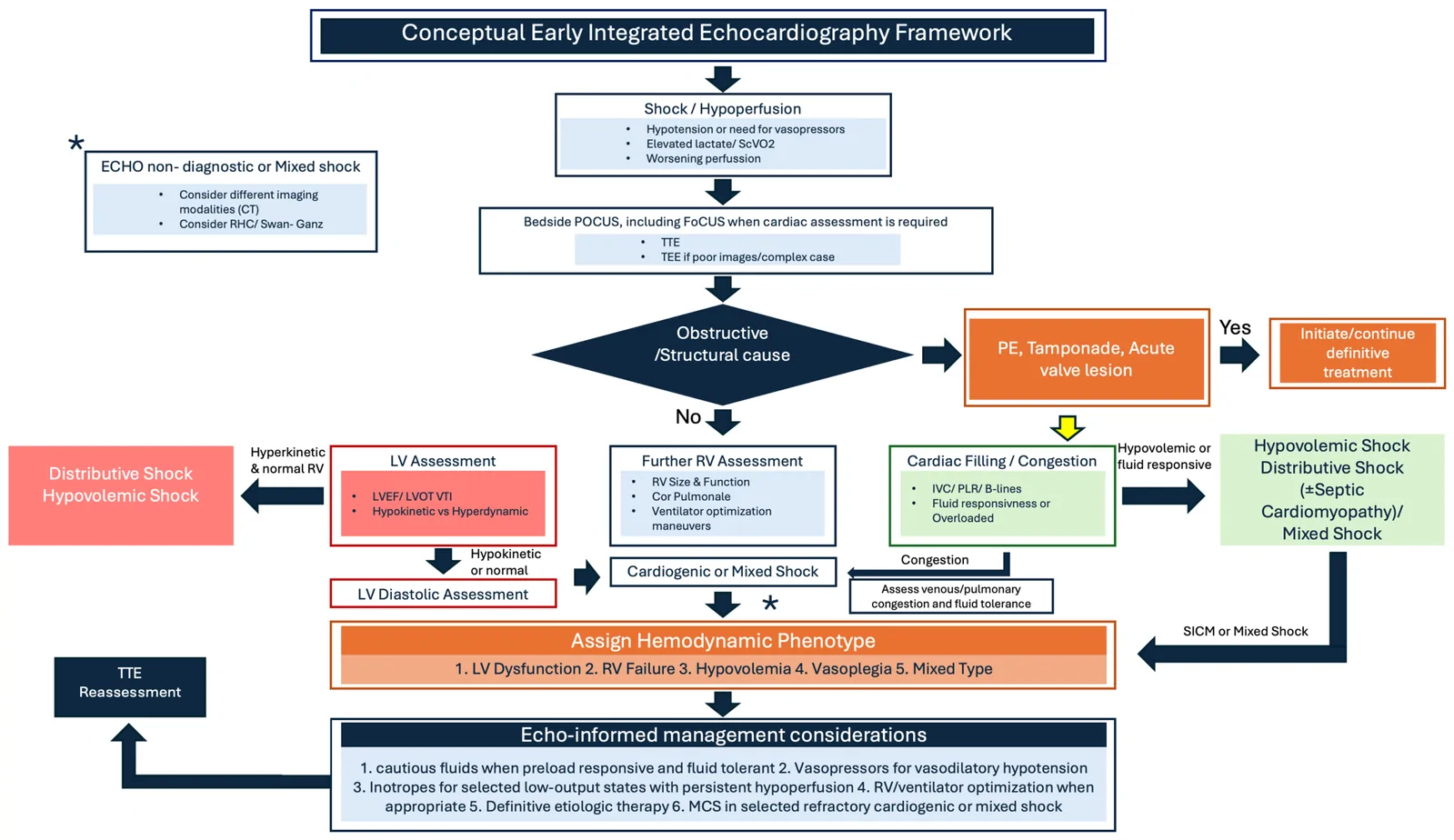
Conceptual early integrated echocardiography framework for the evaluation and management of shock. Echocardiography is integrated concurrently with initial resuscitation and should not delay time-critical etiologic treatment. Point-of-care ultrasound (POCUS), including focused cardiac ultrasound (FoCUS) when cardiac assessment is required, may rapidly identify major obstructive or structural causes and characterize ventricular function, forward flow, loading conditions, and congestion. Regional wall-motion abnormalities may support myocardial ischemia, but their absence does not exclude acute coronary syndrome. The proposed framework is intended for physiological characterization and serial reassessment; individual echocardiographic findings should not be interpreted as validated standalone treatment thresholds. * The asterisk indicates points in the algorithm where echocardiographic assessment alone may be insufficient to fully define the underlying shock phenotype. In these situations—particularly when echocardiographic findings are non-diagnostic or a mixed/cardiogenic shock state remains suspected—additional diagnostic evaluation with complementary imaging and/or invasive hemodynamic assessment (e.g., right-heart catheterization/Swan–Ganz catheterization) may be considered according to the clinical context. (This figure represents a conceptual, physiology-based framework for clinical reasoning and serial reassessment; it has not been prospectively validated as a treatment algorithm and should not supersede established guideline-directed or etiology-specific management). (Abbreviations: ACS, acute coronary syndrome; CO, cardiac output; IVC, inferior vena cava; LV, left ventricle; LVOT VTI, left ventricular outflow tract velocity–time integral; MCS, mechanical circulatory support; PE, pulmonary embolism; POCUS, point-of-care ultrasound; RV, right ventricle; SICM, sepsis-induced cardiomyopathy; TEE, transesophageal echocardiography; TTE, transthoracic echocardiography).

**Figure 3:**
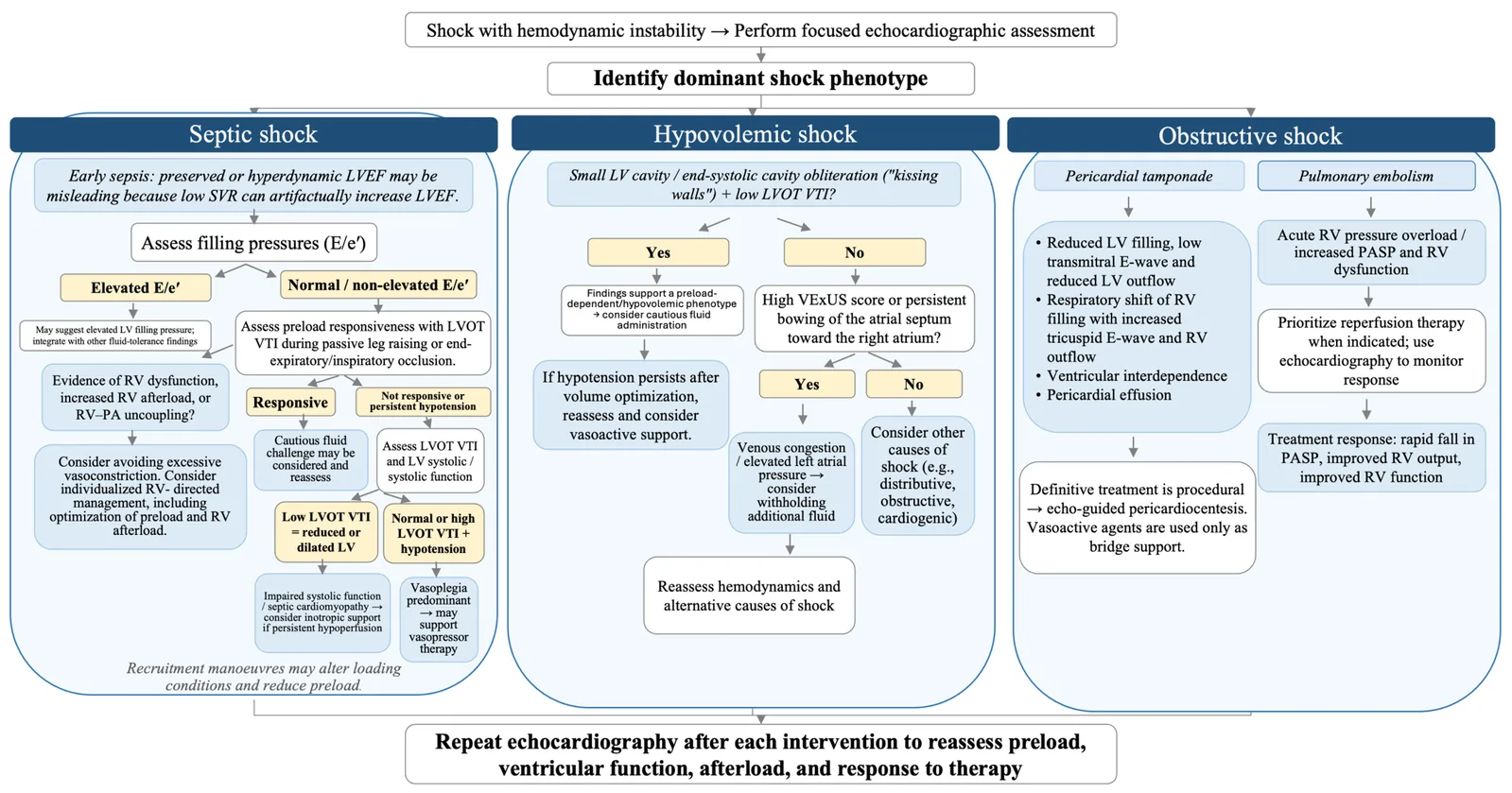
Conceptual echocardiography-based framework for hemodynamic phenotyping and vasoactive management considerations in septic, hypovolemic, and obstructive shock. Focused echocardiography can help characterize the predominant shock phenotype and inform individualized fluid, vasoactive, and procedural management. In septic shock, assessment of filling, preload responsiveness, LVOT VTI, ventricular function, and right ventricular afterload may help distinguish fluid-responsive states, vasoplegia, myocardial dysfunction, and RV dysfunction. In hypovolemic shock, a small LV cavity, end-systolic obliteration, and low LVOT VTI may support fluid resuscitation when consistent with the clinical context, whereas venous congestion or elevated left-sided filling pressures may indicate limited fluid tolerance. In obstructive shock, echocardiography may identify tamponade or pulmonary embolism and facilitate urgent definitive management. Because these parameters are influenced by loading conditions and do not represent validated treatment thresholds, findings should be integrated with the clinical context and serially reassessed to guide individualized therapy and therapeutic response. (This figure represents a conceptual, physiology-based framework for clinical reasoning and serial reassessment; it has not been prospectively validated as a treatment algorithm and should not supersede established guideline-directed or etiology-specific management).

**Figure 4:**
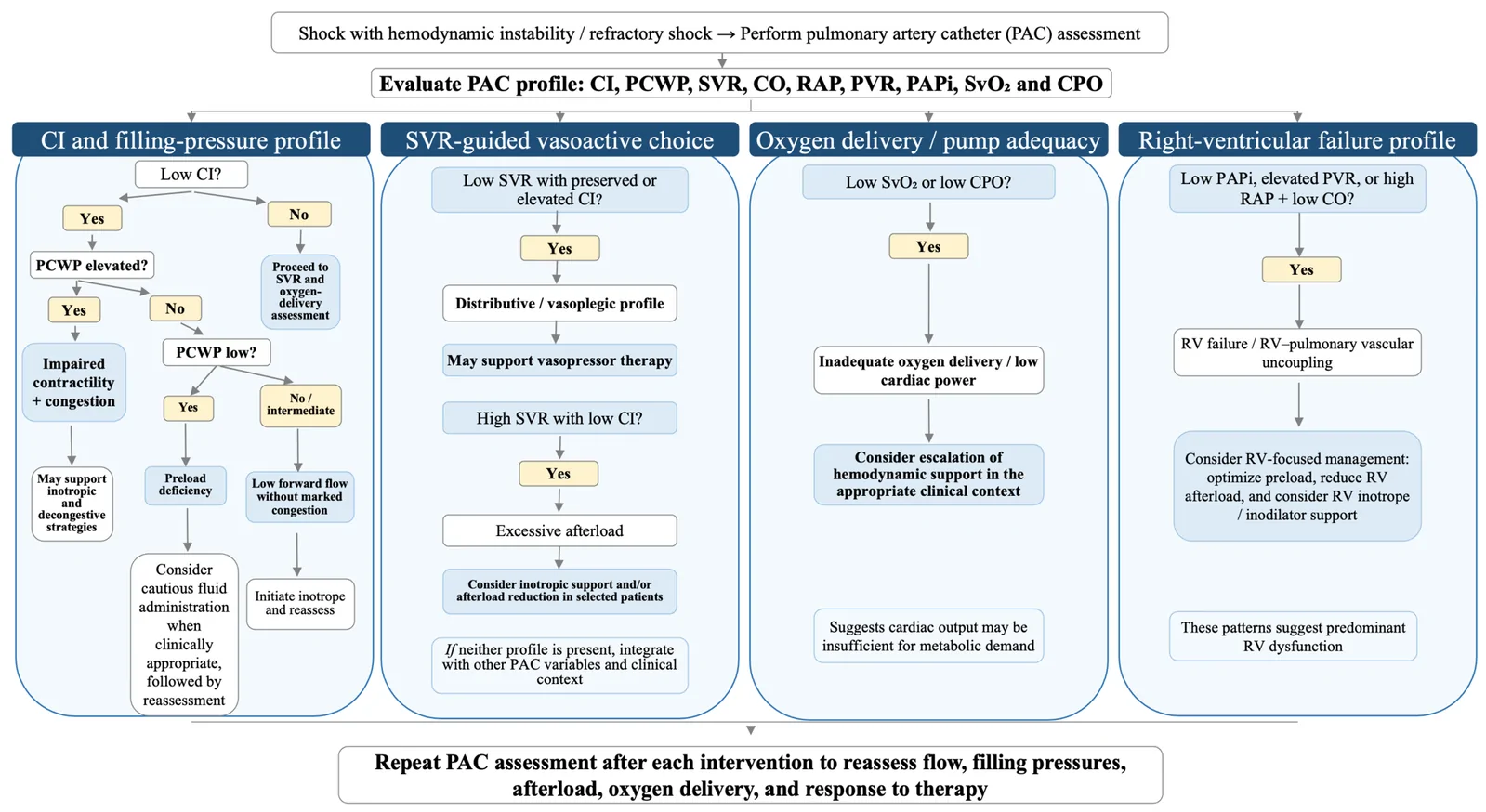
Conceptual PAC-informed hemodynamic framework for selected patients with refractory or complex shock. Pulmonary artery catheterization can provide detailed assessment of cardiac index, filling pressures, systemic and pulmonary vascular resistance, right-sided pressures, mixed venous oxygen saturation, and cardiac power. These variables may help characterize low-flow states, congestion, preload deficiency, vasoplegia, increased afterload, impaired systemic oxygen delivery, and predominant RV dysfunction. Low cardiac index with elevated PCWP may support consideration of inotropic and decongestive strategies, whereas low filling pressures may identify selected patients in whom cautious fluid administration remains appropriate. Low SVR with preserved or elevated cardiac index may support a vasodilatory phenotype, while high SVR with low cardiac index may indicate increased afterload. Low SvO_2_ or cardiac power may suggest inadequate systemic oxygen delivery, and a combination of low PAPi, elevated PVR, elevated RAP, and low cardiac output may support a predominantly RV-related phenotype. These variables should be integrated with clinical and echocardiographic findings and serially reassessed rather than interpreted as standalone treatment thresholds. (This figure represents a conceptual, physiology-based framework for clinical reasoning and serial reassessment; it has not been prospectively validated as a treatment algorithm and should not supersede established guideline-directed or etiology-specific management).

**Table 1:** Etiologies of global left ventricular dysfunction and their clinical and echocardiographic profiles.

Etiology	Findings Supporting the Diagnosis	Findings Arguing Against the Diagnosis	Echocardiographic
Septic Cardiomyopathy	Clinical and laboratory evidence of sepsis, including documented or suspected infection; low systemic vascular resistance; and hyperdynamic or depressed LV function in the appropriate clinical context	Absence of infection; presence of alternative causes of LV dysfunction	Load-dependent and heterogeneous LV phenotype; LVEF may be hyperdynamic/supranormal, preserved, mildly reduced, or markedly depressed depending on loading conditions and disease phase; reduced GLS may reveal systolic impairment despite preserved LVEF; RV dysfunction may coexist; filling pressures may be normal or low early.
Chronic Heart Failure	History of chronic progressive symptoms; treatment with heart failure therapies; structural abnormalities (dilated LV, atrial enlargement, wall thinning or hypertrophy)	Normal cardiac structure; absence of prior symptoms	Dilated HFrEF or preserved EF with HFpEF; left atrial enlargement; abnormal filling patterns (elevated E/e′); possible secondary RV dysfunction
Severe Acidemia	Markedly low pH; temporal association with hemodynamic deterioration; improvement after correction of acidosis	Persistence of LV dysfunction despite normalization of pH	Global hypokinesia, typically reversible; no structural abnormalities; normalization of function after correction of acid–base status
Extensive coronary artery disease	History of coronary artery disease; ECG ischemic changes; possible troponin elevation; supply–demand mismatch	Normal ECG; absence of ischemic history; no biomarker evidence	May present as global hypokinesia or combined regional wall motion abnormalities; reduced LVEF; possible ischemic mitral regurgitation; reduced strain in coronary territories
Acute myocarditis	Recent viral/infectious illness; ECG abnormalities; elevated inflammatory markers; clinical suspicion of myocardial inflammation	Absence of recent infection; findings suggestive of ischemia; lack of inflammatory response	Global or regional hypokinesia; increased wall thickness due to edema; reduced GLS (often disproportionate to LVEF); possible pericardial effusion

Abbreviations: ECG, electrocardiogram; EF, ejection fraction; GLS, global longitudinal strain; HFpEF, heart failure with preserved ejection fraction; HFrEF, heart failure with reduced ejection fraction; LV, left ventricle/left ventricular; LVEF, left ventricular ejection fraction; RV, right ventricle/right ventricular; SVR, systemic vascular resistance.

**Table 2:** Etiologies of segmental wall motion abnormalities and their clinical and echocardiographic profiles.

Etiology	Findings Supporting the Diagnosis	Findings Arguing Against the Diagnosis	Echocardiographic Features
Ischemic Etiology	New regional hypokinesia corresponding to a coronary artery territory; high cardiac biomarkers; ischemic ECG changes	Normal biomarkers; absence of ECG changes; non-coronary perfusion distribution	Segmental wall motion abnormalities in a coronary distribution; reduced LVEF (normal thickness); ischemic mitral regurgitation, when present
Prior Myocardial infarction; Chronic cardiomyopathy of ischemic etiology	History; ECG evidence of prior myocardial infarction;	Absence of prior ischemic history; normal myocardial thickness	Echogenic and thinned myocardial segments; fixed wall motion abnormalities;
Stress-Induced Cardiomyopathy	Preceding emotional or physical stress; discrepancy between extensive WMA and modest biomarker/ECG changes; characteristic early recovery	Significant biomarker elevation proportional to WMA; WMA typical of coronary distribution	Regional WMA not confined to a single coronary territory (e.g., apical ballooning); reversible dysfunction on serial imaging
Myocarditis	Recent infection or inflammatory context; elevated inflammatory markers; possible pericarditis; ECG changes in non-ischemic pattern	Absence of inflammatory signs; strong ischemic pattern	Often inferior/posterior hypokinesia; myocardial edema with increased wall thickness; possible pericardial effusion; reduced GLS
Atypical Septic Cardiomyopathy	Severe sepsis; systemic inflammatory response	A wall-motion pattern strictly confined to a coronary territory with concordant ischemic ECG or biomarker evidence favors a coexisting ischemic process rather than isolated SICM	Regional wall-motion abnormalities may occasionally occur and do not by themselves exclude SICM

Abbreviations: ECG, electrocardiogram; GLS, global longitudinal strain; LVEF, left ventricular ejection fraction; SICM, septic- induced cardiomyopathy; WMA, wall motion abnormalities.

**Table 3:** Right ventricular findings in different shock types.

Condition	RV Findings	Additional Clues
Pulmonary Embolism	RV Dilatation ↑↑ RV Function ↓↓	Acute RV pressure overload; Septal flattening (“D-shape”), McConnell’s sign
Mechanical Ventilation	RV Dilatation ↔ or ↓ RV Function ↓↓	High intrathoracic pressures; improves if PEEP is reduced
Septic Cardiomyopathy	RV Dilatation ↔ RV Function ↔ or ↓	Global myocardial depression; LV also often affected; no pressure/volume overload;

Abbreviations: RV: right ventricle; LV: left ventricle; PEEP: positive end-expiratory pressure. Arrows indicate direction and magnitude of change (↑↑ marked increase; ↓ mild decrease, ↓↓ marked decrease; ↔ no significant change). RV findings should always be interpreted in the context of clinical presentation and hemodynamic status.

**Table 4:** Vasoactive and inotropic agents in shock: predominant mechanisms, typical hemodynamic effects, and selected clinical roles.

Agent	Predominant Pharmacology	SVR	CO/CI	HR	PVR	Selected Clinical Role/Context	Main Cautions
Norepinephrine	α1 > β1 agonist; inopressor	↑↑	↔/↑	↔/↑	Variable, usually ↔/↑	Recommended vasopressor in septic shock; reasonable first vasopressor in hypotensive cardiogenic shock; other vasodilatory states requiring pressure support	Excessive afterload at high doses; peripheral/splanchnic ischemia; arrhythmias possible but less frequent than with dopamine
Epinephrine	α1, β1, β2 agonist; dose-dependent inopressor	Variable; ↑ at higher doses	↑↑	↑↑	Variable	Commonly used in anaphylaxis (IM administration initially); selected refractory septic shock; alternative in selected shock states with myocardial dysfunction	Tachyarrhythmias, increased myocardial oxygen demand, hyperglycemia; β2-mediated lactate elevation may complicate lactate interpretation
Dopamine	D1, β1 and α1 agonism; strongly dose-dependent	Variable → ↑	↑	↑↑	Variable	Limited role; may be considered in selected patients with clinically important bradycardia and low arrhythmic risk or when norepinephrine is unavailable	Higher arrhythmia burden; generally not preferred to norepinephrine in septic shock
Phenylephrine	Pure α1 agonist; vasopressor	↑↑	↔/↓	↔/↓	Variable/↑	Can be used in selected situations in which β-adrenergic stimulation or tachycardia is undesirable; may be considered in dynamic LVOT obstruction	May reduce stroke volume/CO; caution in low-output states and significant RV dysfunction
Vasopressin	V1a-mediated vasoconstriction; non-catecholamine vasopressor	↑↑	↔/↓	↔/↓	Usually less pulmonary than systemic vasoconstriction; effect variable	Adjunct to norepinephrine in septic/vasodilatory shock; catecholamine-sparing strategy	Low-output states; digital, mesenteric or myocardial ischemia, particularly at higher doses
Dobutamine	Predominantly β1 with β2/α1 activity; inodilator	↔/↓	↑↑	↑	↔/↓	Possibly useful for low-output states with persistent hypoperfusion despite adequate filling and perfusion pressure; myocardial or RV dysfunction	Hypotension, tachyarrhythmias, increased myocardial oxygen demand; response may be variable in sepsis
Milrinone	PDE-3 inhibitor; inodilator	↓↓	↑↑	↔/↑	↓	Can be selected in low-output states, particularly with RV–pulmonary vascular dysfunction or reduced β-adrenergic responsiveness	Hypotension, arrhythmias; prolonged effect and accumulation in renal dysfunction
Levosimendan	Calcium sensitizer/inodilator	↓	↑	↔/↑	↓	Possibly useful in acute HF/cardiogenic-shock settings, according to local experience; not routinely recommended in septic shock	Hypotension, arrhythmias, prolonged hemodynamic effect; no established mortality benefit

Hemodynamic effects shown are typical predominant effects and should not be interpreted as fixed responses. They vary with dose, baseline vascular tone, ventricular function, preload, pulmonary vascular load, and concomitant therapies. PVR responses are particularly context-dependent. Clinical roles reflect commonly used or guideline-supported settings rather than absolute indications. Abbreviations: SVR; systemic vascular resistance, CO; cardiac output, HR; heart rate, PVR; pulmonary vascular resistance, LVOT; left ventricular outflow tract, RV; right ventricular, HF; heart failure.

## Data Availability

No new data were created or analyzed in this study. Data sharing is not applicable to this article.

## Abbreviations

The following abbreviations are used in this manuscript:

ACS	Acute coronary syndrome
ALT	Alanine aminotransferase
ARDS	Acute respiratory distress syndrome
AST	Aspartate aminotransferase
ATP	Adenosine triphosphate
BUN	Blood urea nitrogen
CAD	Coronary artery disease
CaO_2_	Arterial oxygen content
CI	Cardiac index
CO	Cardiac output
CPI	Cardiac power index
CPO	Cardiac power output
CRT	Capillary refill time
CvO_2_	Venous oxygen content
DO_2_	Oxygen delivery
DPG	Diastolic pulmonary gradient
Ea	Effective arterial elastance
ECG	Electrocardiogram
Ees	End-systolic elastance
EF	Ejection fraction
FAC	Fractional area change
FoCUS	Focused cardiac ultrasound
GLS	Global longitudinal strain
HF	Heart failure
HFpEF	Heart failure with preserved ejection fraction
HFrEF	Heart failure with reduced ejection fraction
HR	Heart rate
ICU	Intensive care unit
IVC	Inferior vena cava
LA	Left atrium
LV	Left ventricle/left ventricular
LVEDD	Left ventricular end-diastolic diameter
LVEDP	Left ventricular end-diastolic pressure
LVEF	Left ventricular ejection fraction
LVESD	Left ventricular end-systolic diameter
LVOT	Left ventricular outflow tract
LVOTO	Left ventricular outflow tract obstruction
LVOT VTI	Left ventricular outflow tract velocity–time integral
MAP	Mean arterial pressure
MAPK	Mitogen-activated protein kinase
MAPSE	Mitral annular plane systolic excursion
MCS	Mechanical circulatory support
MI	Myocardial infarction
mPAP	Mean pulmonary artery pressure
MR	Mitral regurgitation
NO	Nitric oxide
O_2_ER	Oxygen extraction ratio
PAC	Pulmonary artery catheter
PADP	Pulmonary artery diastolic pressure
PAP	Pulmonary artery pressure
PAPi	Pulmonary artery pulsatility index
PASP	Pulmonary artery systolic pressure
PCWP	Pulmonary capillary wedge pressure
PDE3	Phosphodiesterase-3
PE	Pulmonary embolism
PEEP	Positive end-expiratory pressure
PiCCO	Pulse index continuous cardiac output
PLR	Passive leg raise
POCUS	Point-of-care ultrasound
PvCO_2_	Venous carbon dioxide partial pressure
PVR	Pulmonary vascular resistance
RAP	Right atrial pressure
RV	Right ventricle/right ventricular
RVOT	Right ventricular outflow tract
RVSWI	Right ventricular stroke work index
SBP	Systolic blood pressure
ScvO_2_	Central venous oxygen saturation
SICM	Sepsis-induced cardiomyopathy
STE	Speckle-tracking echocardiography
SV	Stroke volume
SVI	Stroke volume index
SvO_2_	Mixed venous oxygen saturation
SVR	Systemic vascular resistance
TAPSE	Tricuspid annular plane systolic excursion
TAPSV	Tricuspid lateral annular peak systolic velocity
TEE	Transesophageal echocardiography
TPG	Transpulmonary gradient
TPTD	Transpulmonary thermodilution
TTE	Transthoracic echocardiography
V1R	Vasopressin V1 receptor
VExUS	Venous excess ultrasound
VO_2_	Oxygen consumption
VTI	Velocity–time integral
WMA	Wall motion abnormality
